# Are there socioeconomic disparities in women having discussions on human papillomavirus vaccine with health care providers?

**DOI:** 10.1186/1472-6874-12-33

**Published:** 2012-10-04

**Authors:** Ker Yi Wong, Young Kyung Do

**Affiliations:** 1 Program in Health Services and Systems Research, Duke-NUS Graduate Medical School Singapore, 8 College Road, 169857, Singapore

## Abstract

**Background:**

Human papillomavirus (HPV) vaccine recommendation by a health care provider (HCP) is an important predictor of vaccine receipt. We examined whether being of a minority race/ethnicity, having lower income and education, and the lack of health insurance and a regular HCP are each associated with a lower likelihood of a discussion on HPV vaccine occurring between a woman and her HCP.

**Methods:**

A sample of 1,631 women aged 18 years and older was drawn from the 2007 Health Information National Trends Survey. Given that only a subgroup of women who were aware of the HPV vaccine were asked if they had a discussion with their HCPs, we estimated a probit model correcting for sample selection.

**Results:**

Among those aware of the HPV vaccine, 17.3% of respondents reported having discussions on the vaccine with their HCPs. Compared with Whites, African Americans were less likely to be aware of the HPV vaccine but more likely to have discussions with their HCPs concerning the vaccine. A statistically significant association between lower income and education levels and a lower likelihood of HPV vaccine awareness was observed, but low levels of income and education did not appear to affect the probability of having HPV vaccine discussions with HCPs.

**Conclusions:**

Socioeconomically disadvantaged women did not show a lower propensity to have vaccine discussions with their HCPs, suggesting that HCPs can be a major catalyst in increasing vaccine receipt among the higher risk group. The results of the study suggest a two-pronged approach that seeks to raise vaccine awareness among socioeconomically disadvantaged women at the population level and encourages HCPs to intensify discussions about the HPV vaccine with patients.

## Background

Women at high risk of cervical cancer in the United States tend to have less income, less education, and are likely to belong to racial/ethnic minorities, namely African Americans, Hispanics, and Asians [1-4]. This population also bears the highest prevalence of human papillomavirus (HPV) infection [5,6], which accounts for 99% of all cervical cancers [7]; thus, less educated and lower income minority women would likely benefit the most from receiving the HPV vaccine. Unfortunately, HPV vaccination rates among this group are low, and African Americans and Asians are less likely than Whites to be vaccinated against HPV [8]. Low income levels and lack of medical insurance are also associated with lower rates of HPV vaccine initiations [9,10].

The unequal adoption of HPV vaccination can exacerbate the socioeconomic disparities in cervical cancer rates. Many studies have identified predictors of HPV vaccine uptake to address this disparity; most have found HPV vaccine recommendation from a health care provider (HCP) to be an important predictor of vaccine receipt [11-21], both for adult women and for parents considering having their daughters vaccinated. The weight that the public places on vaccine recommendations from HCPs has prompted researchers to suggest that HCPs can help to increase the HPV vaccine uptake rate in the aforementioned population by educating their patients about the vaccine.

Despite the potentially important implications for both clinical and public health practice, research regarding HPV vaccine discussions between patients and their HCPs is currently lacking. This study aimed to identify socioeconomic factors associated with having a patient- or HCP-initiated discussion about the HPV vaccine among a nationally representative sample of American women. As women of lower socioeconomic status are less likely to have adequate access to health care, we postulated that interaction with HCPs follows the same disparate trajectory. Specifically, we hypothesized that being in a minority race/ethnicity, having lower income and education, and the lack of health insurance and a regular HCP are each associated with a lower likelihood of a discussion on HPV vaccine occurring between a woman and her HCP.

## Methods

### Data and sample

The data for this study were drawn from the National Cancer Institute’s 2007 Health Information National Trends Survey (HINTS). The HINTS routinely collects nationally representative data on the US public’s use of and access to cancer-related information, health communication trends and practices, and the public’s perceptions of health risks. HINTS respondents were recruited via two modes: (1) random digit dialing of telephone numbers for recruiting respondents in computer-assisted telephone interviews (CATI) and (2) using mail surveys from a national listing of addresses available from the United States Postal Service. Response rates for the CATI and mail surveys were 24.2% and 31.0%, respectively. Detailed information on the HINTS survey methods can be found elsewhere [22]. Because only CATI included the question that assessed the outcome of interest, this analysis focused on a sub-sample of women recruited for CATI: those who were at least 18 years of age. After excluding observations with missing data, our sample size was 1,631 individuals.

### Outcome variable

The outcome of interest was a binary indicator variable derived from the question, “Has a health care provider such as a doctor or nurse ever talked to you about a cervical cancer vaccine or HPV shot?” This question did not differentiate between a respondent-initiated and a HCP-initiated discussion. Only the respondents who reported having heard of the cervical cancer vaccine or HPV shot before taking part in the survey were asked this question.

### Explanatory variables

This study used socioeconomic and demographic characteristics as explanatory variables. These variables included *household income* (<$20,000; $20,000 to <$50,000; $50,000 to <$75,000; and ≥$75,000), *education* (some high school, high school graduate, some college and college graduate), *race/ethnicity* (White, African American, Hispanic, and other), and *age group* (18–34, 35–39, 40–44, and 45 years and above), as well as *any health care coverage*, and *any regular HCP*. Health care coverage in the HINTS 2007 referred to health insurance, prepaid plans such as health maintenance organizations (HMOs), and government plans such as Medicare. Likewise, HINTS 2007 defined a HCP as a doctor, nurse or other health professional but not a psychiatrist or other mental health professional. For two of these variables – *any health care coverage* and *any regular HCP* – responses of ‘yes’ were coded ‘1’ while responses of ‘no’ were coded ‘0’.

### Statistical analysis

In this study, only women who reported being aware of the HPV vaccine were asked to answer the question on having any discussions with their HCPs about the vaccine. However, HPV vaccine awareness may favor women with more advantageous socioeconomic characteristics. Thus, in terms of having discussions on HPV vaccine with a HCP, a statistical model that only includes respondents who were aware of the vaccine may result in sample selection bias. This issue compromises the study’s internal validity resulting in an underestimation or overestimation of the effect of socioeconomic factors on the likelihood of patients having a discussion on HPV vaccine with their HCPs. External validity is likewise compromised because estimates from the sample selected are not generalizable to the population.

To address sample selection bias, we used the heckprob command in Stata (version 11.0, StataCorp LP), which simultaneously estimates the main probit model of HPV vaccine discussion and the selection model of being aware of the HPV vaccine, while correcting for the potential sample selection bias [23,24].

$$\begin{array}{c} Main\;probit\;model:Discuss^{*}=X\beta_{1}+\varepsilon_{1}\\ Discuss=\{\begin{array}{c} 1\;if\;Discuss^{*}\geq 0\\ 0\;if\;Discuss^{*}<0 \end{array} \end{array}$$

where Discuss* is a latent variable measuring the propensity of a vaccine discussion occurring between a respondent and her HCP and *Discuss* is a dichotomous variable indicating whether or not an HPV vaccine discussion has occurred between a respondent and her HCP. *X* and *β*_*1*_ refer to the matrix of explanatory variables and corresponding coefficients, respectively; *ε*_*1*_ is the error term. A positive coefficient in *β*_*1*_ indicates that the propensity of a woman to have a discussion on the HPV vaccine with her HCP increases with a higher value in the corresponding variable. *Discuss* is only observed when respondents were aware of the HPV vaccine (*Aware* = 1).

$$\begin{array}{c} Selection\;model:Aware^{*}=X\beta_{2}+\varepsilon_{2}\\ Aware=\{\begin{array}{c} 1\;if\;Aware^{*}\geq 0\\ 0\;if\;Aware^{*}<0 \end{array} \end{array}$$

where Aware* is a latent variable measuring the propensity of being aware of the HPV vaccine and *Aware* is a dichotomous variable indicating whether or not a respondent is aware of the HPV vaccine. *X* and *β*_*2*_ respectively denote the same matrix of explanatory variables and corresponding coefficients, and *ε*_*2*_ is the error term. A negative coefficient in *β*_*2*_ indicates that the propensity of a woman to be aware of the HPV vaccine decreases with a higher value in the corresponding variable.

Our model used the correlation coefficient (*ρ*) between *ε*_*1*_ and *ε*_*2*_ to test for sample selection bias. A p<0.05 cutoff was used to determine statistical significance for all analyses. Data were weighted to adjust for survey design.

## Results

### Descriptive statistics

Demographic and socioeconomic characteristics of the study sample are shown in Table 1. The majority of respondents were White, had completed at least high school, and had health care coverage and a regular HCP. The sample was fairly diverse with regard to household income. Among those who were aware of the HPV vaccine, 17.3% reported having had a discussion about the HPV vaccine with their HCPs. The percentages of respondents who had HPV vaccine discussions with their HCPs did not show discernible trends with increasing household income or education levels. The group with the highest percentage of respondents who reported having had HPV vaccine discussions with their HCPs were African Americans (26.6%), followed by Hispanics (17.2%), Whites (15.8%) and other (13.7%). Among 81.5% of the respondents who were aware of the HPV vaccine, Whites had the highest percentage of vaccine awareness (88.7%), followed by African Americans (73.2%), other (66.6%), and lastly, Hispanics (58.8%). The percentage of respondents who were aware of the HPV vaccine was higher among respondents with higher household income than those from lower income groups (92.4% versus 55.3%). Similarly, HPV vaccine awareness was higher among respondents with higher (versus lower) education levels (89.7% versus 47.1%).

**Table 1:** Summary statistics of study sample

	**Whole sample**		***Selection indicator:***		***Main outcome:***	
			**Aware of HPV vaccine**		**Discussed with HCP on HPV vaccine**	
	**N**	**Column %**	**N positive**	**Row %**	**N positive**	**Row %**
Overall	1631		1314	81.5	161	17.3
Age group						
18–34	236	30.7	204	86.4	52	26.0
35–49	108	10.1	98	86.6	16	16.5
40–44	116	10.0	98	81.9	24	29.6
45 and above	1171	49.2	914	77.3	69	8.7
Race/ethnicity						
White	1282	66.9	1084	88.7	117	15.8
African American	127	14.9	90	73.2	19	26.6
Hispanic	133	11.9	78	58.8	13	17.2
Other	89	6.3	62	66.6	12	13.7
Household income						
$75,000 or more	492	30.0	453	92.4	75	21.0
$50,000 to <$75,000	303	18.9	259	89.2	24	15.9
$20,000 to <$50,000	537	34.6	434	80.3	44	14.3
<$20,000	299	16.5	168	55.3	18	17.5
Education						
College graduate	613	27.9	549	89.7	72	18.1
Some college	475	33.0	407	90.0	51	17.8
High school graduate	407	27.3	301	77.6	30	14.6
Less than high school	136	11.8	57	47.1	8	20.8
Has any insurance						
No	160	14.2	106	64.0	17	14.7
Yes	1471	85.8	1208	84.4	144	17.6
Has any regular HCP						
No	251	21.4	178	73.0	24	14.8
Yes	1380	78.6	1136	83.8	137	17.9

All frequencies are unweighted and all percentages are weighted. *HPV*=human papillomavirus; *HCP*=health care provider.

### Probit model of HPV vaccine discussion with HCP, with correction for sample selection

Results of the probit model analysis of HPV vaccine discussions with HCPs (Table 2) showed that African American women (coefficient 0.515, p<0.05) were more likely than White women to report having such discussions. On the other hand, women aged 45 years and above (coefficient −0.386, p<0.05) were less likely to have discussions on HPV vaccine with their HCPs than women in the reference age group of 18–34 years. The level of household income and education, as well as having insurance and a regular HCP did not show statistically significant associations with patients having discussions on HPV vaccine with their HCPs.

**Table 2:** **Probit model of having HPV vaccine discussion with HCP, correcting for sample selection**^**a**^

**Variables**	***Selection equation:***		***Main equation:***	
	**Aware of HPV vaccine**		**Discussed with HCP on HPV vaccine**	
	**Coefficient**	**(95% C.I.)**	**Coefficient**	**(95% C.I.)**
Age group				
18–34	Reference		Reference	
35–39	0.119	(−0.385, 0.623)	−0.290	(−0.638, 0.059)
40–44	−0.373	(−0.868, 0.122)	0.178	(−0.182, 0.538)
45 and above	−0.630**	(−1.024,–0.235)	−0.386*	(−0.726,–0.045)
Race/ethnicity				
White	Reference		Reference	
African American	−0.505**	(−0.859,–0.152)	0.515*	(0.114, 0.917)
Hispanic	−0.745**	(−1.147,–0.343)	0.233	(−0.182, 0.649)
Other	−1.028**	(−1.550,–0.506)	0.369	(−0.148, 0.885)
Household income				
$75,000 or more	Reference		Reference	
$50,000 to <$75,000	−0.278	(−0.605, 0.050)	−0.132	(−0.455, 0.191)
$20,000 to <$50,000	−0.360*	(−0.634,–0.086)	−0.158	(−0.519, 0.202)
<$20,000	−0.839**	(−1.125,–0.552)	0.199	(−0.135, 0.533)
Education				
College graduate	Reference		Reference	
Some college	0.197	(−0.079, 0.474)	−0.063	(−0.345, 0.220)
High school graduate	−0.279*	(−0.534,–0.024)	0.026	(−0.326, 0.377)
Less than high school	−0.743**	(−1.139,–0.347)	0.471	(−0.006, 0.947)
Has any insurance				
No	Reference		Reference	
Yes	0.152	(−0.249, 0.553)	−0.088	(−0.624, 0.447)
Has any regular HCP				
No	Reference		Reference	
Yes	0.200	(−0.083, 0.482)	0.114	(−0.260, 0.488)
Constant	1.800**	(1.255, 2.346)	−0.563	(−1.175, 0.049)
Correlation (*ρ*)			−0.845 **	(−0.969, –0.387)

^a^Sample size=1,631; *HPV*=human papillomavirus; *HCP*=health care provider; *p<0.05, **p<0.01.

Analysis of the selection model revealed that – compared with the reference group (i.e., 18–34 years old, White, household income above $75,000, college graduates) – women aged 45 years and above, belonging to an ethnic minority group, with household income below $50,000 or with education levels lower than or equivalent to high school were less likely to be aware of the HPV vaccine.

The statistically significant correlation coefficient of the probit model (−0.845, p<0.01, Table 2) suggested that the model would suffer from sample selection bias if the study sample only consisted of respondents who were aware of the HPV vaccine. In other words, unexplained factors (*ɛ*_*2*_) in the model of awareness of HPV vaccine were correlated with unexplained factors (*ɛ*_*1*_) that predict discussion with HCPs. The negative sign indicates that *ɛ*_*1*_ and *ɛ*_*2*_ are negatively correlated.

## Discussion

The primary goal of this study was to examine socioeconomic differences in the likelihood of a discussion on HPV vaccine occurring between a woman and her HCP using a model that accounts for potential sample selection bias. Our analysis suggested that African American women were *more* likely to have had discussions about the HPV vaccine with their HCPs than White women. Overall, we did not find evidence that socioeconomic disadvantages were associated with a lower chance of having a discussion on HPV vaccine with HCPs. A similar finding was reported by Hughes et al. (2009), who did not find evident socioeconomic disparities in the odds of reporting HCPs as previous sources of HPV vaccine information [25].

These results are encouraging although not intuitively predictable. Many studies demonstrate that the socioeconomically disadvantaged tend to have less access to health care [26-28], which leads to the assumption that such individuals may have fewer opportunities to discuss the HPV vaccine with their HCPs, supposing they have one. However, our results contradict this assumption; the influence of a patient’s race and socioeconomic status on the screening practices of HCPs could primarily explain this contradiction. Studies have shown that HCPs are more likely to provide counseling services on the prevention of sexually transmitted infections (STIs) to a patient from a minority ethnic group and a lower socioeconomic status than one from a non-minority ethnic group [29] and a higher socioeconomic status [30], respectively. HCPs generally prioritize their screening practices based on actual epidemiological data, showing the association of race and socioeconomic status with STIs [31]. Since HPV infection is a STI, a similar reasoning can be used to explain the lack of socioeconomic disparities observed in this study. A HCP who is aware that the prevalence of HPV infections is higher among African American women and women with low levels of education and income will be more likely to discuss the HPV vaccine with these groups.

Secondly, both the psychosocial factors that affect a woman’s behavior on seeking health information and the predictors of a HCP recommending the HPV vaccine influence whether a woman discusses the HPV vaccine with her HCP. Individual psychosocial factors such as having a higher internal locus of control [32,33] and a preference for involvement in health-related decision making [34] were found to contribute positively to information seeking behavior. Trust in HCPs was also correlated with more information seeking from HCPs [35]. Among HCPs, an early adopter of a new intervention generally has a higher intention of recommending the HPV vaccine [36,37]. Physicians’ practices [38,39] and their beliefs and attitudes towards the HPV vaccine [37,40] were also found to affect their propensity to recommend the HPV vaccines to their patients. Albeit beyond the scope of this study, the presence of numerous HCP- and patient-related factors, including their intricate interaction with one another, possibly mitigated the socioeconomic disparities in having discussions on HPV vaccine with HCPs.

However, similar to other studies [9,25,26,41,42], our statistical analysis has revealed a troubling disparity in HPV vaccine awareness across multiple dimensions of socioeconomic status. Belonging to a minority race/ethnicity, and having a lower household income and education level were independently associated with a lower probability of being aware of the HPV vaccine. The disparities in HPV vaccine awareness were all statistically significant and showed a fairly consistent gradient for both income and education.

The results of this study have a number of important implications for research, practice and policy. In terms of research, our statistical approach provides a fuller picture of socioeconomic disparities in HPV vaccine by simultaneously examining two related indicators. However, the eventual uptake of the vaccine depends on a complex interplay of factors in a woman’s psychosocial milieu as well as other demographic and socioeconomic factors. Thus, future research that assesses the impact of socioeconomic status on the integrated continuum of HPV vaccine awareness, acquisition of information, and vaccine uptake will greatly inform policy makers and health care providers in guiding education and practice.

Regarding the implications for clinical practice, HCPs may be the preferred source of health care information for the socioeconomically disadvantaged because of the lack of other reliable options; this preference results in a higher likelihood of having discussions on HPV vaccine. Thus, HCPs can play a pivotal role in dispensing accurate, objective information about the vaccine, and help dispel any myths or negative attitudes about HPV vaccination, thereby allowing socioeconomically disadvantaged women to make informed decisions for themselves and for their daughters.

Lastly, our findings that lower socioeconomic status is associated with lower awareness but not with lower opportunities for discussions on HPV vaccine with HCPs has important public health and policy implications. Primarily, social disparities in HPV vaccine uptake will be greatly reduced if discussions between patients and their HCPs become more prevalent. Targeted outreach programs should also be promoted in order to raise awareness about the vaccine among socioeconomically disadvantaged women and should be tailored to address attitudes and perceptions that are specific to each ethnic group. In addition, given the potential for HCPs to ameliorate social disparities in HPV vaccine uptake and its importance, public health efforts can be directed toward giving full support to HCPs for HPV vaccination promotion. This support can come in the form of informative pamphlets in various vernaculars to enhance communication and provide easily accessible, timely, and up-to-date information on HPV vaccine research.

Notwithstanding these implications, the findings presented herein should be considered in the context of a few limitations. A key limitation of this study was the short interval between the FDA approval of the quadrivalent HPV vaccine in 2006 and the launch of the HINTS in early 2008. Thus, the small number of respondents who reported having HPV vaccine discussions with their HCPs may not be reflective of the corresponding number today. Future research involving HCPs who are more familiar with the vaccines may offer a more accurate picture of the prevalence of vaccine discussions between women and their HCPs. A second limitation, which is related to the first, is that four years have elapsed since the launch of HINTS in early 2008; thus, the results may not reflect the current situation today. However, while average awareness of the vaccine may have increased over the years, the increment of increase is unknown and its rate differs among populations. Hence, this study is still pertinent in underserved populations whose awareness of the HPV vaccine and accessibility to HCPs remains low, and in which identifying ways to best target patient education remains crucial. Additionally, the self-reported status of having discussions on HPV vaccine with a HCP is subject to recall bias. Moreover, the content and extent of the discussion was unknown, and a standard definition for what qualifies as a “discussion on HPV vaccine” was not described in the survey. This could have resulted in an underestimation or overestimation of the number of respondents who reported having discussions on HPV vaccine with their providers. Lastly, the HINTS was not developed to include constructs that aim to capture HCP factors and individual psychosocial factors that could have influenced the likelihood of women having discussions on HPV vaccine with their HCPs. Future research surveys designed to capture all of these factors could give a more comprehensive analysis of socioeconomic disparities in HPV vaccine discussions.

## Conclusions

Numerous studies have demonstrated that socioeconomically disadvantaged women are at higher risk of HPV-related mortality and morbidity. However, the comparable tendencies to have discussions on HPV vaccine with HCPs amongst women across different socioeconomic groups differed from the wide disparities in awareness found in our study, suggesting that HCP involvement in broader public health efforts is of paramount importance in reducing socioeconomic disparities in HPV infection and cervical cancer.

## Competing interests

The authors declare that they have no competing interests.

## Authors’ contributions

KYW conceived of the study, analyzed the data, drafted and completed the manuscript. YKD supervised data analysis and helped with revisions. All authors read and approved the final manuscript.

## Pre-publication history

The pre-publication history for this paper can be accessed here:

http://www.biomedcentral.com/1472-6874/12/33/prepub
